# Efficient CD44-targeted magnetic resonance imaging (MRI) of breast cancer cells using hyaluronic acid (HA)-modified MnFe_2_O_4_ nanocrystals

**DOI:** 10.1186/1556-276X-8-149

**Published:** 2013-04-02

**Authors:** Taeksu Lee, Eun-Kyung Lim, Jaemin Lee, Byunghoon Kang, Jihye Choi, Hyo Seon Park, Jin-Suck Suh, Yong-Min Huh, Seungjoo Haam

**Affiliations:** 1 Department of Chemical and Biomolecular Engineering, Yonsei University, Seoul, 120-749, South Korea; 2 Department of Radiology, College of Medicine, Yonsei University, Seoul, 120-752, South Korea; 3 YUHS-KRIBB Medical Convergence Research Institute, Seoul, 120-752, South Korea; 4 Severance Biomedical Science Institute, Seoul, 120-752, South Korea; 5 Department of Architectural Engineering, Yonsei University, Seoul, 120-749, South Korea

**Keywords:** Colloidal nanoparticles, Nanomedicine, Nanobioimaging, Hyaluronic acid, Magnetic resonance imaging

## Abstract

Targeted molecular imaging with hyaluronic acid (HA) has been highlighted in the diagnosis and treatment of CD44-overexpressing cancer. CD44, a receptor for HA, is closely related to the growth of cancer including proliferation, metastasis, invasion, and angiogenesis. For the efficient detection of CD44, we fabricated a few kinds of HA-modified MnFe_2_O_4_ nanocrystals (MNCs) to serve as specific magnetic resonance (MR) contrast agents (HA-MRCAs) and compared physicochemical properties, biocompatibility, and the CD44 targeting efficiency. Hydrophobic MNCs were efficiently phase-transferred using aminated polysorbate 80 (P80) synthesized by introducing spermine molecules on the hydroxyl groups of P80. Subsequently, a few kinds of HA-MRCAs were fabricated, conjugating different ratios of HA on the equal amount of phase-transferred MNCs. The optimized conjugation ratio of HA against magnetic content was identified to exhibit not only effective CD44 finding ability but also high cell viability through *in vitro* experiments. The results of this study demonstrate that the suggested HA-MRCA shows strong potential to be used for accurate tumor diagnosis.

## Background

CD44 is a cell-surface glycoprotein antigen of breast cancer cells that is well known for its specific binding with hyaluronic acid (HA) [1-3]. It is the multifunctional cell-surface molecule involved in pathologic properties of cancer cells such as cell proliferation, differentiation, migration, angiogenesis, and chemokines [4-6]. Therefore, detecting CD44 is vital for accurate diagnosis as well as identifying effective anticancer drugs. Especially, when HA binds with CD44, this binding-mediated signals trigger cytological activities such as structural changes in the membrane and tumor cell migration [7-10]. Hence, HA has been frequently utilized as a targeting moiety to detect CD44 in the diagnosis and treatment of specific cancers directly associated with CD44 [11-14].

For sensitive and specific detection of cancer via the CD44 receptor, molecular imaging has been strongly considered due to the accurate acquisition of highly sensitive images and deeper insight into *in vivo* conditions [15-17]. Of the various molecular imaging techniques, magnetic resonance (MR) imaging has been widely recommended because it is non-invasive and provides high-resolution and tomographic real-time images at the cellular and molecular levels [18-20]. Therefore, CD44-targeted MR imaging has been applied in the treatment of cancer such as monitoring therapeutic efficacy and determining the progonosis of cancer.

To facilitate better interpretation of the MR images, recently developed MnFe_2_O_4_ nanocrystals (MNCs), synthesized by the thermal decomposition method in the organic phase, are well suited because of their fine crystalline structure and high magnetic sensitivity with an excellent size and composition control [21,22]. However, these MNCs are insoluble in aqueous phase and could not be used directly in biomedical applications, so appropriate modification is necessary for phase transfer as well as further conjugation of specific targeting moieties such as HA [23,24]. In addition, identification of specific amounts of targeting moieties on the MNCs resulted in the most efficient cellular uptake and imaging *in vitro*[25]. Therefore, finding the optimal HA density on MNCs is needed for the most effective diagnosis and treatment for CD44-overexpressed breast cancer.

**Figure 1:**
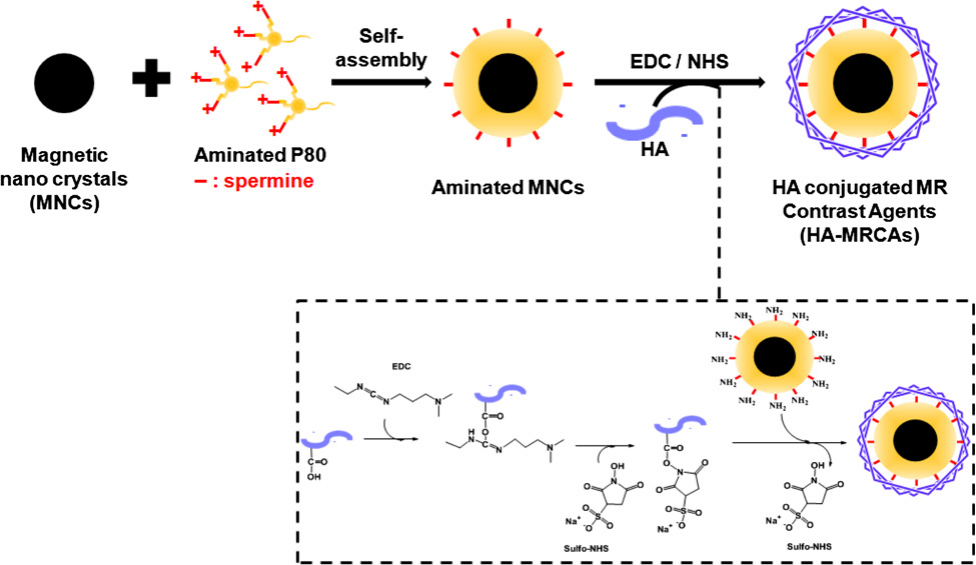
Schematic illustration of the synthesis of HA-conjugated MR contrast agents.

Herein, we report the development of HA-modified MR contrast agents (HA-MRCAs) for utilization in the efficient targeted detection and diagnosis of CD44-overexpressing cancer via MR imaging. Water-soluble aminated MNCs (A-MNCs) were firstly formulated via the nano-emulsion method. To investigate the optimal amount of HA for CD44 targeting with high efficiency, HA-MRCAs were prepared by conjugating different amounts of HA molecules to the A-MNCs (Figure 1). HA-MRCAs preserved colloidal stability and represented CD44 targeting ability as well as enhanced cell viabilities due to the modification with HA. The physicochemical properties and biocompatibilities of HA-MRCAs were fully characterized, and their enhanced sensitivity with selective binding to the CD44-abundant cancer cells was comparatively investigated via MR imaging.

## Methods

### Materials

Polysorbate 80 (polyoxyethylene sorbitan monooleate, P80), spermine, 1,10-carbonyldiimidaziole (CDI), 1,4-dioxane (99.8%), iron(III) acetylacetonate, manganese(II) acetylacetonate, 1,2-hexadecanediol, dodecanoic acid, dodecylamine, benzyl ether, and 1-ethyl-3-(3-dimethylaminopropyl)-carbodiimide (EDC) were purchased from Sigma-Aldrich Chemical (St. Louis, MO, USA). Hyaluronic acid (20 kDa) was obtained from Lifecore Biomedicals (Chaska, MN, USA). Phosphate-buffered saline (PBS; 10 mM, pH 7.4), Dulbecco's modified Eagle's medium, Roswell Park Memorial Institute medium (RPMI), and fetal bovine serum (FBS) were purchased from Gibco (Life Technologies, Carlsbad, CA, USA). Both MDA-MB-231 and MCF-7 cells, breast carcinoma cell lines [26-28], were obtained from the American Type Culture Collection (Manassas, VA, USA). Sulfo-*N*-hydroxysuccinimide (sulfo-NHS) and 2,4,6-trinitrobenzene sulfonic acid (TNBSA) solution were purchased from Pierce (Thermo Scientific, Waltham, MA, USA). All other chemicals and reagents were of analytical grade.

### Synthesis of MNCs

Monodispered magnetic nanocrystals, soluble in hydrophobic solvent, were synthesized using the thermal decomposition method [21]. First, iron(III) acetylacetonate (2 mmol), manganese (II) acetylacetonate (1 mmol), 1,2-hexadecanediol (10 mmol), dodecanoic acid (6 mmol), and dodecylamine (6 mmol) were dissolved in 20 mL of benzyl ether under a blanket of nitrogen. The mixture was reacted for 2 h at 200°C and then further heated at 300°C for 1 h. All processes were under nitrogen atmosphere. After the mixtures were cooled at room temperature, the products were purified twice with 20 mL of pure ethanol. Finally, MNCs were grown to approximately 12 nm by the seed-mediated growth method.

### Preparation of A-MNCs and HA-MRCAs

A-MNCs were fabricated using the nano-emulsion method [23]. First, 10 mg of MNCs was dissolved in 4 mL of *n*-hexane (organic phase). The organic phase was injected into 30 mL of de-ionized water (aqueous phase) containing 100 mg of aminated P80. After mutual saturation, the solution was emulsified for 20 min under ultrasonification (ULH700S, Ulssohitech, Cheongwon-gun, South Korea) at 450 W. The mixture was kept overnight at room temperature to remove the volatile organic solvent. The products were purified using a centrifugal filter (Centriprep YM-3, 3-kDa molecular weight cutoff (MWCO), Amicon, Millipore Corporation, Billerica, MA, USA) in triplicate at 3,000 rpm for 30 min.

HA-MRCAs with different molar ratios of HA were fabricated by EDC-NHS chemistry. First, the pH of the A-MNC solution was adjusted to neutral condition by the addition of 0.1 N HCl solution. Then, various amounts of HA (0.43, 1.7, and 6.8 μmol) were dissolved in the 40 mL of de-ionized water followed by the addition of EDC and sulfo-NHS. Each HA solution was added to A-MNC solution containing 5 mg of MNCs. The HA and A-MNCs were reacted for 2 h at room temperature. Finally, EDC, sulfo-NHS, and unbound HA were removed using dialysis (MWCO, 25, 000) against excess de-ionized water.

### Characterization of A-MNCs and HA-MRCAs

The size distributions and zeta potential values of A-MNCs and HA-MRCAs were measured using laser scattering (ELS-Z, Otsuka Electronics, Osaka, Japan). The inorganic ratios (%) and the crystallinities of magnetic nanocrystals in A-MNCs and HA-MRCAs were analyzed using a thermo-gravimetric analyzer (SDT-Q600, TA Instruments, Newcastle, DE, USA) and X-ray diffraction (X-ray diffractometer Ultima3, Rigaku, Tokyo, Japan) at 25°C, respectively. The magnetic properties of A-MNCs and HA-MRCAs were also detected by a vibration sample magnetometer (model 9407, Lake Shore Cryotronics, Inc., Westerville, OH, USA) at 25°C.

### Cell viability assay for A-MNCs and HA-MRCAs

The cytotoxic effect of A-MNCs and HA-MRCAs against MDA-MB-231 cells (CD44-abundant cancer cell line) was analyzed by measuring the inhibition of cell growth using an assay for WST-1 ((4-(3-(4-lodophenyl)-2-(4-nitrophenyl)-2H-5-tetrazolio)-1,3-benzene disulfonate)). MDA-MB-231 cells were maintained in RPMI containing 10% FBS and 1% antibiotics at 37°C in a humidified atmosphere with 5% CO_2_. MDA-MB-231 cells were harvested at a density of 1.0 × 10^4^ cells/100 μL in a 96-well plate and incubated at 37°C in 5% CO_2_ atmosphere overnight. The cells were then treated with various concentrations of A-MNCs and HA-MRCAs for 24 h. After incubation, the cells were rinsed with 100 μL PBS (pH 7.4, 1 mM), and then 10 μL of WST-1 solution was added to each well. The absorbance was measured at 450 nm with a reference wavelength of 600 nm. The relative percentage of cell viability was determined as the ratio of formazan intensity in viable cells treated with A-MNCs and HA-MRCAs to the intensity in non-treated (control) cells [29-31].

### Cellular targeting efficiency of HA-MRCAs

The targeting efficiency of HA-MRCAs was examined by MR imaging of breast carcinoma cell line MDA-MB-231 cells (high CD44 expression) and MCF-7 cells (low CD44 expression). First, target cells (1.0 × 10^7^ cells) were harvested and washed three times with blocking buffer (FBS (0.2%) and NaN_3_ (0.02%) in phosphate-buffered solution (pH 7.4, 10 mM)) to inhibit non-specific binding effects. The solutions containing HA-MRCAs were applied to each cell line (1 and 0.5 μg, respectively) at 4°C for 30 min. The cells were then washed with blocking buffer three times to remove non-binding HA-MRCAs. Next, 200 μL of 4% paraformaldehyde was added to re-suspend the cells. After targeting efficiency was analyzed via MRI, the cells were dissolved in nitric acid for 2 h at 180°C, and the concentrations of magnetic nanocrystals (Fe + Mn) were measured using inductively coupled plasma atomic emission spectrometry (ICP-AES).

### MR imaging procedures

We performed *in vitro* MR imaging experiments with a 1.5-T clinical MRI instrument with a micro-47 surface coil (Intera, Philips Medical Systems, Best, The Netherlands). The *T*2 weights of the A-MNC- and HA-MRCA-treated cells (MDA-MB-231 and MCF-7 cells) were measured by the Carr-Purcell-Meiboom-Gill (CPMG) sequence at room temperature with the following parameters: TR = 10 s, 32 echoes with 12-ms even echo space, number of acquisitions = 1, point resolution of 156 × 156 μm, and section thickness of 0.6 mm. For acquisition of *T*2-weighted MR images of A-MNC- and HA-MRCA-treated cells, the following parameters were adopted: resolution of 234 × 234 μm, section thickness of 2.0 mm, TE = 60 ms, TR = 4,000 ms, and number of acquisitions = 1.

## Results and discussion

### Characterization of aminated P80

MNCs, soluble in non-polar organic solvent with high monodispersity, were made using the thermal decomposition method to serve as MR contrast agents. For the identification of optimal HA density for efficient CD44-overexpressed breast cancer cell imaging and phase transference of hydrophobic MNCs into aqueous phase, the tri-hydroxyl groups of polysorbate 80 (P80) were modified with amine groups using spermine and the cross linker, 1,1^′^-carbonyldiimidazole (CDI) [32]. CDI was used to activate hydroxyl groups of P80 and generate reactive imidazole carbamate intermediates. When the amine group of spermine attacked the intermediate, imidazoles were released, and stable tri-urethane (*N*-alkyl carbamate) linkages were fabricated. After conjugation, the characteristic bands of aminated P80 were verified by FT-IR spectra, which represented N-H stretching of an amine group (3,550 cm^−1^), C-N stretching of an amide group (3,400 cm^−1^), and N-H bending of an amine group (1,600 cm^−1^) (Additional file 1: Figure S1). On the contrary, the O-H stretching of hydroxyl groups (3,500 cm^−1^) of P80 was not detected in aminated P80 because all hydroxyl groups of P80 reacted with excess CDI. Furthermore, the chemical structures of aminated P80 were analyzed by ^1^H-NMR to show *δ* values of 7.11 (−CONH-), 4.29 (−NH_2_), 3.22 (−OCH_2_-), 2.72, 1.77 (−CH_2_-), and 2.17 (−NH-) ppm (Additional file 1: Figure S2). To quantify the primary amine groups (−NH_2_) in aminated P80, a TNBSA assay was used since primary amine groups replace sulfonic acid groups in TNBS molecules. Therefore, this substitution produces a chromogenic complex for which the absorbance at 355 nm is proportional to the number of amine groups (Additional file 1: Figure S3) [33]. A standard curve was created using glycine because this amino acid molecule possesses one primary amine group per molecule. The absorbance of aminated P80 confirmed that the number of primary amine groups in aminated P80 was approximately 2.4-fold higher than that of glycine. These results showed that all hydroxyl groups of P80 were modified with amine groups, and the MNCs could be modified with HA through the generation of an amide bond.

### Synthesis and characterization of A-MNCs and HA-MRCAs

Subsequently, A-MNCs were fabricated with pre-synthesized aminated P80 through the nano-emulsion method. The HA, CD44-targeting polysaccharide, was conjugated to the A-MNCs by EDC/NHS chemistry to provide breast cancer cell affinity. Carboxylic acid groups in HA were activated by EDC, and then sulfo-NHS was reacted to generate sulfo-NHS ester. Amine groups as nucleophiles on the A-MNCs were conjugated with these activated ester groups, and the NHS group rapidly left the intermediates, thereby creating stable amide linkages between A-MNCs and HA to form HA-MRCAs [34].

**Figure 2:**
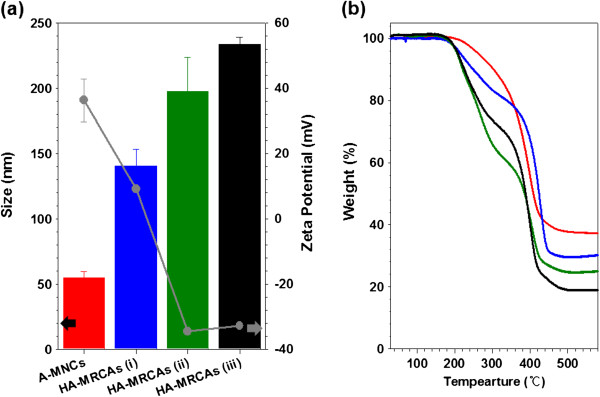
**Average size, zeta potential values, and thermo-gravimetric analysis.** (**a**) The average size (bar graph) and zeta potential values (gray circle) and (**b**) the thermo-gravimetric analysis of A-MNCs and HA-MRCAs: A-MNCs (red), HA-MRCAs (i) (blue), HA-MRCAs (ii) (green), and HA-MCRAs (iii) (black).

**Figure 3:**
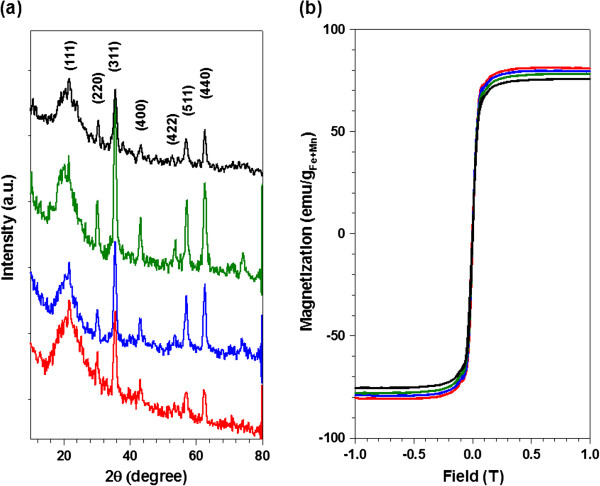
**X-ray diffraction patterns and magnetic hysteresis loops.** (**a**) XRD patterns and (**b**) magnetic hysteresis loops of A-MNCs and HA-MRCAs with insertion of the main crystalline phases of magnetic nanocrystals: A-MNCs (red), HA-MRCAs (i) (blue), HA-MRCAs (ii) (green), and HA-MCRAs (iii) (black).

Various HA-MRCAs were prepared by changing the amount of HA to equal that of A-MNCs (HA-MRCAs (i) 4.4 × 10^−1^ μmol, HA-MRCAs (ii) 1.7 μmol, HA-MRCAs (iii) 7.0 μmol and A-MNCs were fixed to MNCs of 5 mg) for comparing the targeting efficiency with respect to the amount of HA. Their average sizes were measured using light scattering (A-MNCs, 54.9 ± 4.6 nm; HA-MRCAs (i), 140.5 ± 12.6 nm; HA-MRCAs (ii), 197.8 ± 26.3 nm; HA-MRCAs (iii), 233.8 ± 5.2 nm). As expected, the size of HA-MRCAs proportionally increased with increasing amount of conjugated HA (Figure 2a) due to the increase in the organic layer, and this was also confirmed by thermogravity measurement (Figure 2b). Light scattering represented that both A-MNCs and HA-MRCAs were also well dispersed in the aqueous phase without aggregation because of the steric hindrance by hydrogen bonding with the biocompatible polymer HA and aminated P80 on the coating layer of nanoparticles and water. It was also confirmed by TEM images (Additional file 1: Figure S4) [1,22]. The surface charge of A-MNCs was strongly positive (36.3 ± 6.6 mV) due to the abundant amine groups. HA-MRCAs (i) revealed a weak positive charge (9.16 ± 0.9 mV) owing to the remaining amine groups, whereas HA-MRCAs exhibited a negative charge (HA-MRCAs (ii), −34.5 ± 1.0 mV; HA-MRCAs (iii), −32.8 ± 0.5 mV) because aminated surfaces were covered with the carboxlic groups of HA (Figure 2a). We examined the colloidal stability of A-MNCs and HA-MRCAs against various pH conditions (4~10) and NaCl concentrations (0~1.0 M), followed by physiological pH and NaCl conditions, after mixing overnight at room temperature (Additional file 1: Figure S4). Both A-MNCs and HA-MRCAs (HA-MRCA (i), HA-MRCA (ii), and HA-MRCA (iii)) exhibited sufficient colloidal stability without aggregation under these conditions. These results assessed that A-MNCs and HA-MRCAs are highly potent to serve as MR contrast agents [35-39]. Though MNCs were encapsulated with organic compounds, A-MNCs and HA-MRCAs preserved the crystallinities of MNCs, as demonstrated by the characteristic X-ray diffraction (XRD) patterns at 2Θ values of 30.3° (220), 35.8° (311), 43.6° (400), 57.5° (511), and 62.7° (440), corresponding with the mixed spinel structure (Figure 3a) [40]. To assess the potential of using HA-MRCAs in MR probe applications, sensitivities to the magnetic field were confirmed. Regardless of phase transfer with aminated P80 and HA conjugation, A-MNCs and HA-MRCAs exhibited superparamagnetic properties without a hysteresis loop (Figure 3b), and their saturation magnetization values were similar and approximately 80.0 emu/g_Fe+Mn_. Therefore, they could be used as contrast agents of MR imaging.

### Relaxivity of HA-MRCAs and A-MNCs

To assess the MR contrast effect of HA-MRCAs, we performed MR imaging using HA-MRCAs, with A-MNCs used as a control. The relaxivity coefficients were measured and calculated (A-MNCs 361.6 mM^−1^ s^−1^, HA-MRCAs (i) 380.0 mM^−1^ s^−1^, HA-MRCAs (ii) 366.0 mM^−1^ s^−1^, and HA-MRCAs (iii) 407.3 mM^−1^ s^−1^), and representative *T*2-weighted MR images were collected (Additional file 1: Figure S5). HA-MRCAs exhibited MR contrast effects that were remarkably higher than those of commercial MR imaging contrast agents (ferumoxide 190.5 mM^−1^ s^−1^) based on the fact that HA-MRCAs induced better contrast in MR imaging than ferumoxide [41]. The high relaxivity coefficients of HA-MRCAs were achieved not only by the substitution of one of the Fe ions with a Mn ion, but also by the high crystallinity and monodispersity of the MNCs synthesized by the thermal decomposition method [42-44]. Further, relaxation rate (*R*2) and the concentration of MNCs had a linear relationship in all HA-MRCAs. Therefore, the targeting efficiency of HA-MRCAs could be determined based on the concentration of the MR contrast agent in the tumor, which should be directly proportional to the relaxivity. Interestingly, HA-MRCAs exhibited similar or better relaxivity compared with A-MNC. This might be attributed to the HA domain of HA-MRCAs. HA can form many hydrogen bonds with surrounding water molecules owing to its abundant functional groups, such as hydroxyl and carboxylic groups. Hydrogen bonding between HA in the coating layer of HA-MRCAs and water molecules formed the hierarchical structures. In this structure, the mobility of water molecules in the diffusing layer is confined, and the residence time of water increases due to hydrogen bonding. These phenomena result in the enhancement of the transverse relaxation rate [45-51]. Therefore, HA-MRCAs possessed similar relaxivity, even after HA modification.

### Cell viability assay with A-MNCs and HA-MRCAs

**Figure 4:**
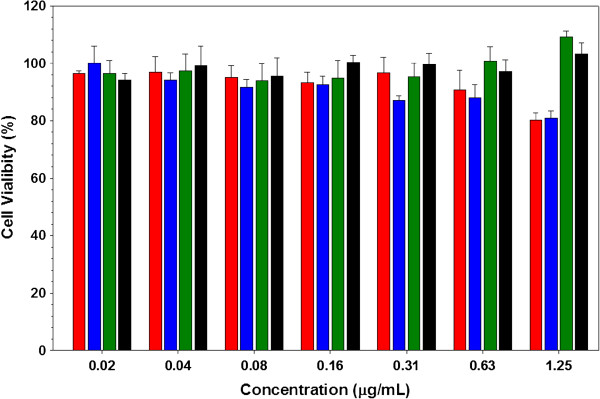
**Cell viabilities of MDA-MB-231 cells.** The cells were treated with various concentrations of A-MNCs and HA-MRCAs: A-MNCs (red), HA-MRCAs (i) (blue), HA-MRCAs (ii) (green), and HA-MCRAs (iii) (black).

As shown in Figure 4, the cellular toxicity values of A-MNCs and HA-MRCAs were examined in target cancer cells (MDA-MB-231: high CD44 expression) varied with concentrations (2.0 × 10^−2^~1.25 μg/mL) for 24 h using a cell proliferation kit. Both A-MNCs and HA-MRCAs were found to be highly non-toxic, based on the fact that there was greater than 80% cell viability without an inhibitory effect on proliferation or growth in the MDA-MB-231 cells. In particular, HA-MRCAs (ii) and HA-MRCAs (iii) revealed lower cytotoxicity compared to A-MNCs and HA-MRCAs (i) at high concentration (1.25 μg/mL). This is due to the positive surface charges of A-MNCs and HA-MRCAs (i), which induced disruption and solubilization of cell membranes by electrostatic interaction [52,53].

### Targeting efficiency of HA-MRCAs against CD44-overexpressing cancer cells

**Figure 5:**
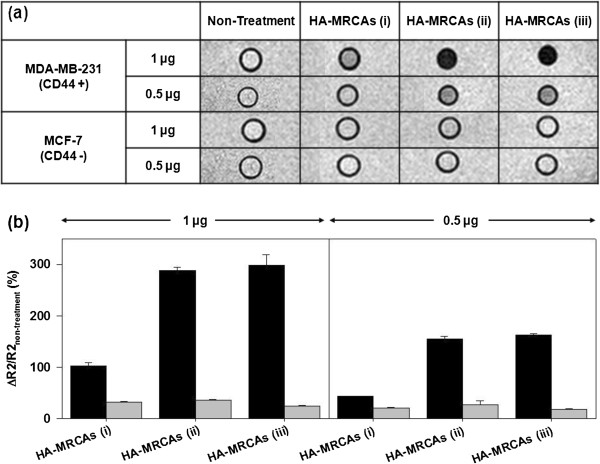
**MR images and graph of Δ *R *2/ *R *2**_**non-treatment**_**.** (**a**) *T*2-weighted MR images and (**b**) the graph of Δ*R*2/*R*2_non-treatment_ of MDA-MB-231 (black bar) and MCF-7 (gray bar) after HA-MRCA treatment versus untreated cells at 1 and 0.5 μg of metal (Fe + Mn) concentrations.

**Figure 6:**
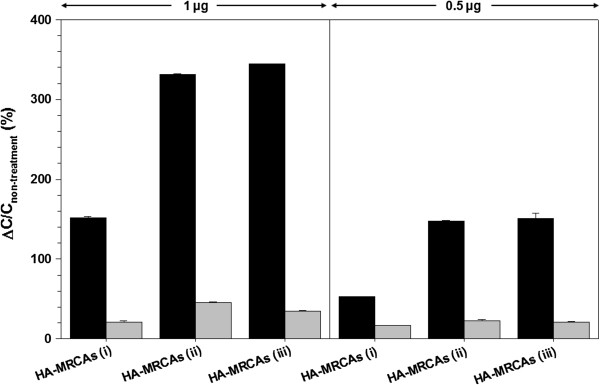
**Relative concentrations.** The relative concentrations (%) of MDA-MB-231 (black bar) and MCF-7 (gray bar) after HA-MRCA treatment versus untreated cells at 1 and 0.5 μg of metal (Fe + Mn) concentrations using ICP-AES analysis.

To compare the detection efficiency of CD44 according to the amount of HA, we investigated the targeted MR contrast ability of HA-MRCAs against MDA-MB-231 (CD44 overexpressed) and MCF-7 (CD44 less expressed) [22,26-28,54]. *T*2-weighted MR images of HA-MRCA-treated cells were confirmed, and their MR signal intensity ratio, which indicates the relaxation rate (*R*2) difference between HA-MRCA-treated cells and non-treated cells (Δ*R*2/*R*2_Non-treatment_, where Δ*R*2 = *R*2 − *R*2_Non-treatment_ and *R*2 = *T*2^−1^), were fitted in the MR images (Figure 5a). Strong dark MR images and a high relaxivity difference represented the efficient targeting ability of HA-MRCAs. In the case of HA-MRCAs (i), a surface charge shift from positive to neutral and insufficient amount of HA conjugation on the A-MNCs resulted in the weak targeting ability of HA-MRCAs (i), as shown in MR images and signal results (1 and 0.5 μg of HA-MRCAs (i)-treated MDA-MB 231 cells, 102.3 ± 7.6% and 43.8 ± 0.6%; 1 and 0.5 μg of HA-MRCAs (i)-treated MCF-7 cells, 32.7 ± 1.3% and 20.7 ± 1.9%). On the contrary, HA-MRCAs (ii) and HA-MRCAs (iii), which bound more HA than HA-MRCAs (i), revealed strong black signals in MR images of MDA-MB-231 cells compared with those of MCF-7 cells due to specific binding between CD44 and HA of HA-MRCAs. In addition, these results also revealed that HA-MRCAs (ii) and HA-MRCAs (iii) had more efficient targeting efficiency than HA-MRCAs (i) because more HA was conjugated (1 μg of HA-MRCAs (ii)- and HA-MRCAs (iii)-treated MCF-7 cells, 36.9 ± 1.0% and 24.5 ± 1.7%; 0.5 μg of HA-MRCAs (ii)- and HA-MRCAs (iii)-treated MCF-7 cells, 26.8 ± 8.4% and 18.3 ± 1.0%; 1 μg of HA-MRCAs (ii)- and HA-MRCAs (iii)-treated MDA-MB-231 cells, 288.4 ± 6.2% and 297.9 ± 20.5%; 0.5 μg of HA-MRCAs (ii)- and HA-MRCAs (iii)-treated MDA-MB-231 cells, 155.3 ± 5.3% and 162.7 ± 3.0%) (Figure 5b). Using ICP-AES, we analyzed the MNC (Fe + Mn) concentrations in the cells (MDA-MB-231 and MCF-7 cells) after treatment with HA-MRCAs, and this tended to correspond with MR signal intensity (Figure 6). Consequently, from the targeting efficacy experiments of HA-MRCAs against CD44-abundant cancer cells, HA-MRCAs (ii) and HA-MRCAs (iii) showed similar detection efficiencies even though fourfold more HA was used to fabricate the HA-MRCAs (iii). Based on these experiments, the ability to target CD44 did not differ when the CD44 amount was higher than the amount of HA in HA-MRCAs (ii).

## Conclusion

HA-MRCAs with various ratios of HA were fabricated to determine the most efficient conditions for achieving accurate detection of CD44-overexpressing cancer. With HA conjugation, the surface charge changed from positive to negative, resulting in an increase in cell viability. Then, we confirmed that HA-MRCAs exhibited similar relaxivity in spite of the HA modification, which allowed the comparison of targeting efficiency via MR imaging. Varying the HA ratio could control the targeting ability of each HA-MRCA. Especially, HA-MRCAs (ii) and HA-MRCAs (iii) represented a sufficiently high MR imaging sensitivity to diagnose CD44-overexpressing cancer from *in vitro* studies. HA was modified four more times in the fabrication of HA-MRCAs (iii) compared to HA-MRCAs (ii); however, both HA-MRCAs (ii) and HA-MRCAs (iii) revealed similar targeting ability. Based on these results, HA-MRCAs (ii) appear to be appropriate for diagnosis of CD44-overexpressing cancer and can be utilized as drug carriers as well as in theragnosis systems using HA [55].

## Abbreviations

A-MNCs: Aminated MNCs; HA: Hyaluronic acid; HA-MRCAs: HA-modified MR contrast agents; MNCs: Nagnetic nanocrystals; MRI: Magnetic resonance imaging; P80: Polysorbate 80

## Competing interests

The authors declare that they have no competing interests.

## Authors’ contributions

TL performed the experiments, suggested the scheme, and drafted the manuscript. EKL guided the idea and the experiments and checked the scheme and figures. JL revised it critically for important intellectual content. BK performed experiments. JC reviewed the scheme and contents. HSP, JSS, and YMH supervised the project. SJH tailored the idea, finalized the manuscript, and has given the final approval of the version to be published. All authors read and approved the final manuscript.

## Supplementary Material

Additional file 1 **Supporting information.** A pdf file showing the synthesis and characterization of aminated P80, colloidal stability test, TEM detection, and MR imaging procedures of A-MNcs and HA-MRCAs. Click here for file
