# Supplementary material for: Efficient CD44-targeted magnetic resonance imaging (MRI) of breast cancer cells using hyaluronic acid (HA)-modified MnFe2O4 nanocrystals
Source: Nanoscale Res Lett. 2013 Apr 2;8(1):149. doi: 10.1186/1556-276X-8-149 (PMC3621698; doi:10.1186/1556-276X-8-149)
Supplement: Additional file 1 — Supporting information. A pdf file showing the synthesis and characterization of aminated P80, colloidal stability test, TEM detection, and MR imaging procedures of A-MNcs and HA-MRCAs. [file 1556-276X-8-149-S1.pdf]

**Supporting Information**

# Efficient CD44-targeted magnetic resonance imaging (MRI) of breast cancer cells using hyaluronic acid (HA)-modified $MnFe_2O_4$ nanocrystals

Taeksu Lee[1], Eun-Kyung Lim[2,3], Jaemin Lee[1], Byunghoon Kang[1], Jihye Choi[1], Hyo Seon Park[5],Jin-Suck Suh[2,3,4], Young-Min Huh[2,3,4,*], Seungjoo Haam[1,3,*]

[1] *Department of Chemical and Biomolecular Engineering, Yonsei University, Seoul 120-749, South Korea*

[2] *Department of Radiology, College of Medicine, Yonsei University, Seoul 120-752, South Korea*

[3] *YUHS-KRIBB Medical Convergence Research Institute, Seoul 120-752, South Korea*

[4] *Severance Biomedical Science Institute, Seoul 120-752, South Korea*

[5] *Department of Architectural Engineering, Yonsei University, Seoul 120-749, South Korea*

**S1. Synthesis and characterization of aminated polysorbate 80 (aminated P80)**

Aminated P80 was synthesized as previously describe [1]. Polysorbate 80 (1.5 mmol) and CDI (4.5 mmol) were added to 60 mL of 1,4-dioxane and magnetically stirred for 1 h. After the reactants were fully dissolved, spermine (4.5 mmol) was added to the solution, and the mixture was stirred for an additional 24 h at room temperature. A rotary evaporator was used to remove extra solvent. 10 mL of de-ionized water was added to dissolve the remaining aminated P80. The solution containing aminated P80 was dialyzed (MWCO 1000) against excess de-ionized water to purify the product. After dialysis, the aminated P80 was lyophilized. The synthesized aminated P80 was analyzed by Fourier-transform infrared spectroscopy (FT-IR, Varian, Excalibur™ series) and $^1$H NMR (400 MHz, Bruker NMR spectrometer) using $CDCl_3$ as the solvent. The primary amine groups of aminated P80 were quantified using the TNBS (2,4,6-trinitrobenzene sulfonic acid) assay. Various concentrations (6.25 ~ 100 μM) of aminated P80 were prepared with 0.1 M sodium bicarbonate buffer at pH 8.5 for quantifying the primary amine groups of aminated P80. Each aminated P80 solution (0.5 mL) and 0.01 % (w/v) TNBS solution (0.25 mL) were mixed and incubated at 37 ˚C for 2 h. Then, 1 % sodium dodecyl sulfate (0.25 mL) and 1 N hydrochloric acid (0.125 mL) were added. Glycine was also tested in a similar manner for a control experiment. The absorbance of aminated P80 and glycine solutions at 355 nm were analyzed using a UV-Vis spectrometer (Optizen 2120UV, MECASYS Co.)

**S2. Colloidal Stability Test of A-MNCs and HA-MRCAs**

The colloidal stability of A-MNCs and HA-MRCAs against various pH conditions and NaCl concentrations were tested from their resistance to NaCl and pH induced aggregation after

mixing for over-night at room temperature[2]. The stability of A-MNCs and HA-MRCAs against diverse NaCl and pH conditions was detected by light scattering.

**S3. Transmission Electron Microscope (TEM) detection of A-MNCs and HA-MRCAs**

Transmission electron microscopy (TEM, JEM-1011, JEOL, Japan) at an acceleration voltage of 80 kV was used to obtain TEM images of A-MNCs and HA-MRCAs

**S4. MR imaging procedures of A-MNCs and HA-MRCAs solutions**

We performed *in vitro* MR imaging experiments with a 1.5 T clinical MRI instrument with a micro-47 surface coil (Intera; Philips Medical Systems, Best, The Netherlands). The T2 weights of the A-MNCs and HA-MRCAs solutions were measured by the Carr-Purcell-Meiboom-Gill (CPMG) sequence at room temperature with the following parameters: TR 10 s, 32 echoes with 12 ms even echo space, number of acquisitions 1, point resolution of 156 x 156 μm, and section thickness of 0.6 mm.

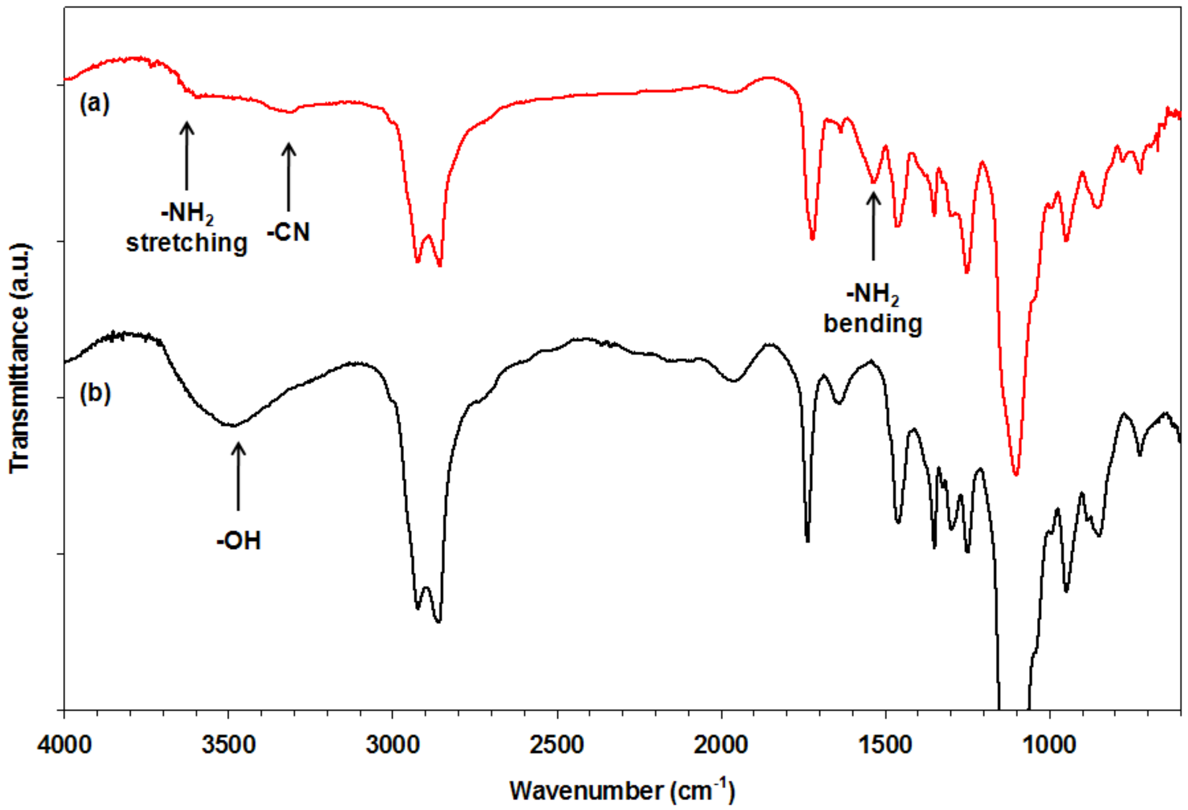


**Fig. S1** FT-IR spectra of (a) aminated P80 (red line) and (b) P80 (black line).

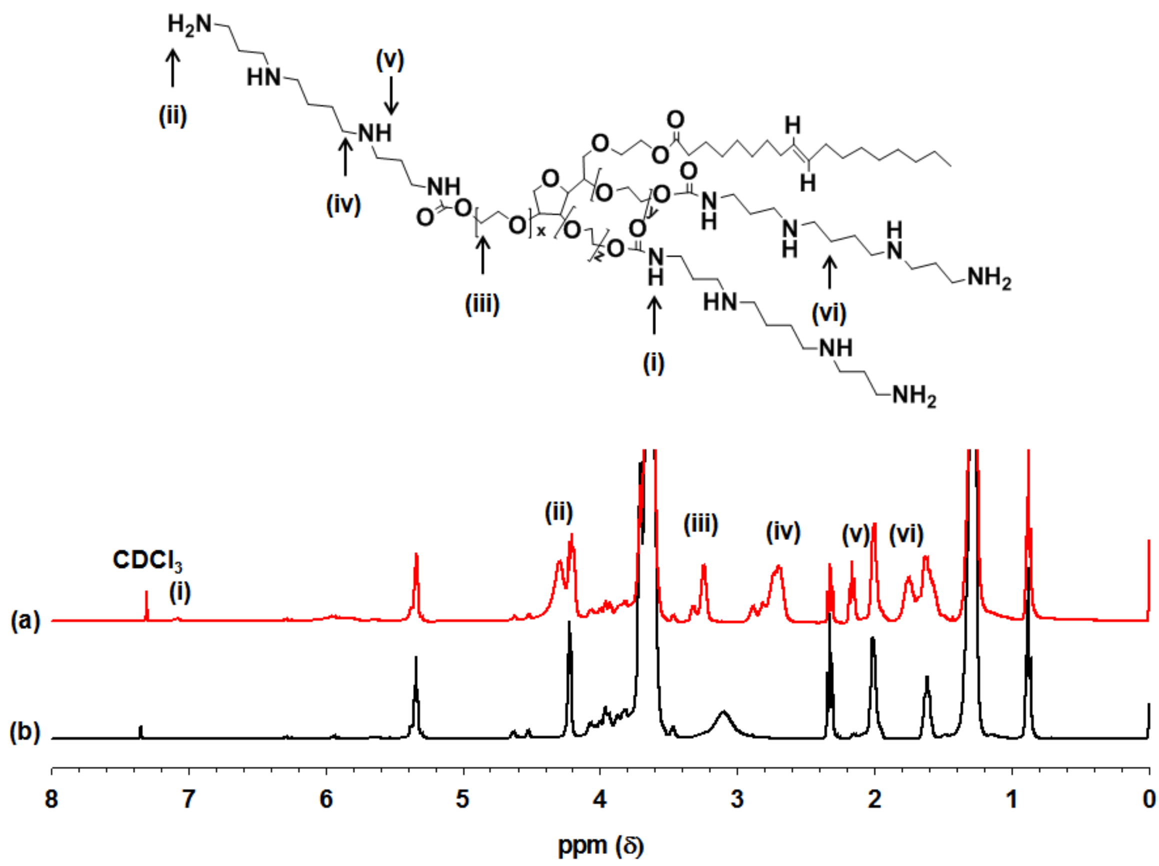


**Fig. S2** $^1$H-NMR spectra of (a) aminated P80 (red line) and (b) P80 (black line).

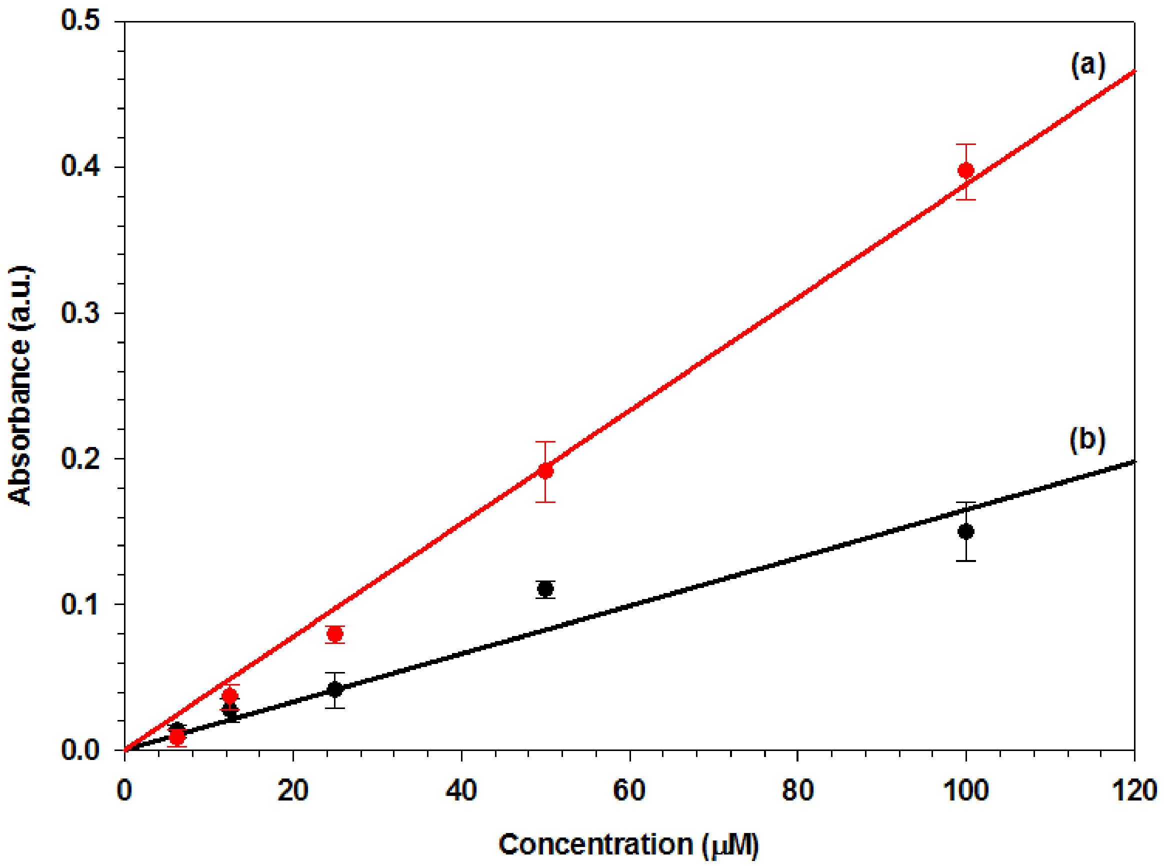


**Fig. S3** The quantitative analysis of amine groups for (a) aminated P80 (red line) and (b) glycine (black line) using TNBS assay.

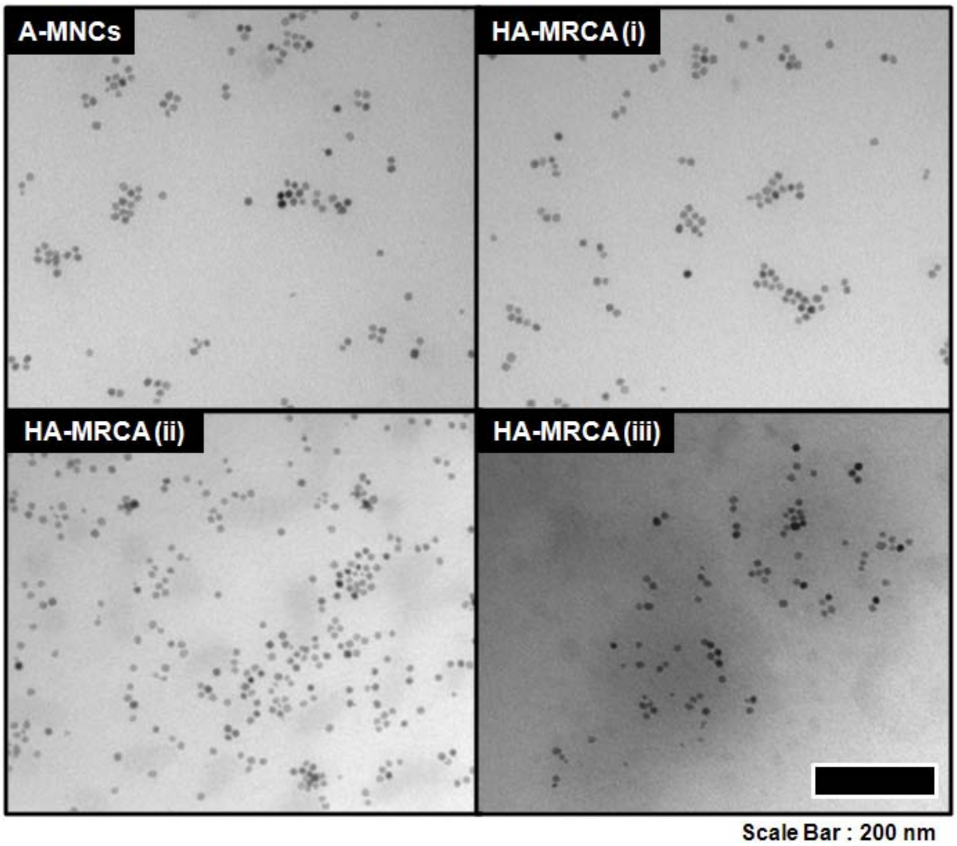


**Fig. S4.** TEM images of A-MNCs, HA-MRCA (i), HA-MRCA (ii) and HA-MRCA (iii).

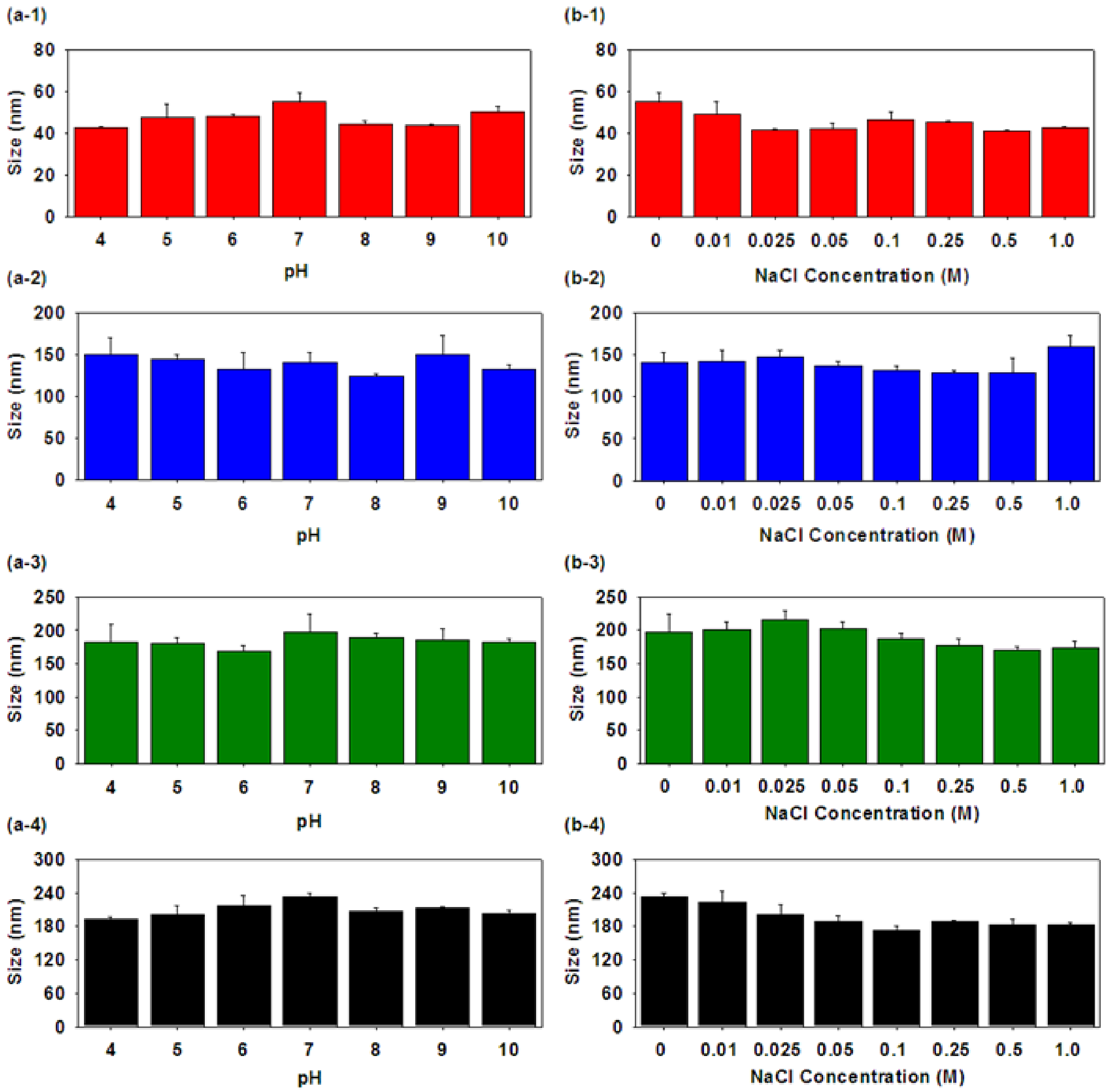


**Fig. S5** The colloidal stability of A-MNCs and HA-MRCAs against various pH condition (4 ~ 10) and NaCl concentration (0 ~ 1.0 M) for overnight at room temperature (a: pH stability test, b: NaCl stability, 1: A-MNCs, 2: HA-MRCAs (i), 3: HA-MRCAs (ii) and 4: HA-MRCAs (iii)).

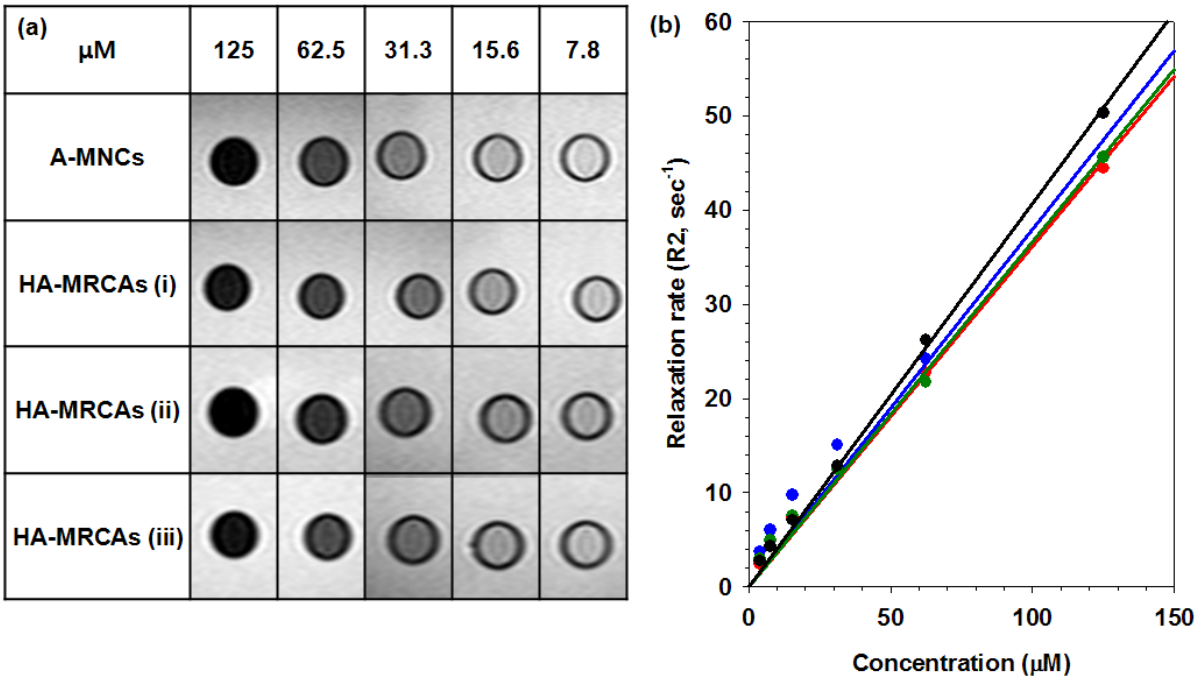


**Fig. S6** (a) T2-weighted MR images and (b) relaxation rate (R2) graph versus various concentrations of A-MNCs and HA-MRCAs: A-MNCs (red); HA-MRCAs (i) (blue); HA-MRCAs (ii) (green); HA-MRCAs (iii) (black).

References


1. Lim EK, Yang J, Suh JS, Huh YM, Haam S: **Synthesis of Aminated Polysorbate 80 for Polyplex-Mediated Gene Transfection.** *Biotechnol Progr* 2010, **26:**1528-1533.
2. Seo SB, Yang J, Lee ES, Jung Y, Kim K, Lee SY, Kim D, Suh JS, Huh YM, Haam S: **Nanohybrids via a polycation-based nanoemulsion method for dual-mode detection of human mesenchymal stem cells.** *J Mater Chem* 2008, **18:**4402-4407.
